# Intraocular silicone oil brain migration associated with severe subacute headaches: a case report

**DOI:** 10.1186/s40942-020-00273-6

**Published:** 2021-02-15

**Authors:** Thiago José Muniz Machado Mazzeo, Gabriel Almeida Veiga Jacob, Paulo Henrique Horizonte, Henrique Monteiro Leber, André Marcelo Vieira Gomes

**Affiliations:** Retina and Vitreous Department, Suel Abujamra Institute, Tamandaré, 693 – Liberdade, 01525-001 São Paulo, Brazil

**Keywords:** Retinal detachment, Victrectomy, Silicone oil, Radiology

## Abstract

**Purpose:**

The aim of this article is to report a rare case in which a patient presented symptomatic silicon oil brain migration, documented by MRI, several years after vitreoretinal surgery.

**Methods:**

This is a case report with a prospective literature review.

**Patients:**

The patient described in the case report.

**Results:**

Case report.

**Discussion/conclusions:**

For several years, silicone oil (SiO) has been widely used as a long-term intravitreal tamponading agent to treat complex retinal detachments. There are rare reports in the literature demonstrating the migration of SiO into the brain. The aim of this article is to report a rare case in which the patient presented severe headaches several years after vitreoretinal surgery, with migrated SiO appearing in MRI as an oval lesion within the horn of the right lateral ventricle. To the best of our knowledge, there are very few reports of symptomatic SiO brain migration in the literature.

## Main text

### Introduction

Silicone oil (SiO) was first described as an intraocular tamponade for retinal detachment in 1962 by Cibis and colleagues [1]. Since then, it has been widely used as a long-term intravitreal tamponading agent to treat complex retinal detachments. Many intraocular complications of SiO have been reported, including cataract formation, oil emulsification with secondary glaucoma, and subretinal oil migration [2, 3].

Infiltration of SiO into the retrolaminar optic nerve was first demonstrated pathologically by Ni et al. in 1983 [4]. Posteriorly, rare reports showed SiO migration and its progression through the optic chiasm and brain [5, 6]. The exact mechanism of why it occurs is not yet fully understood. Various factors might play a role in physical silicone oil migration, such as congenital anatomical deformities (optic pit), long-term elevated intraocular pressure, degeneration of the optic nerve, and migration of phagocytosed emulsified oil bubbles by macrophages [7].

Although it is a rare complication, SiO migration is described in the literature as a benign and non-symptomatic incidental radiographic finding [8]. We report a case in which the patient presented severe headaches several years after vitreoretinal surgery with SiO tamponade. To the best of our knowledge, there are very few reports of symptomatic SiO brain migration in the literature.

### Case report

A 67 years old white woman related severe headaches, dating from one month ago. She had no other symptoms and no family history of migraines or glaucoma as well. A pars plana vitrectomy in OD with SiO tamponade (5000 cSt) was performed in 2016 due to a tractional-rhegmatogenous retinal detachment caused by proliferative diabetic retinopathy. The patient evolved with intraocular hypertension and glaucoma in the same eye, being later submitted to a trabeculectomy (2018) and to a cyclophotocoagulation in 2019.

The patient had a vision of no-light perception in OD and 20/50 in OS (+ 3.00 −1.75 × 90). On the slit-lamp biomicroscopy examination of OD, the patient was pseudophakic with a significant posterior capsule opacification, while the left eye was unremarkable. The intraocular pressure was 20\16 mmhg by *Goldmann’s* applanation tonometry. The posterior segment examination showed the presence of SiO and a total excavation of the optic disc with intense optic disk pallor in OD. There was an inferior retinectomy associated with subretinal PVR next to inferior vascular arcades. In the left eye, there was a physiological optic disc cupping and a PDR.

**Fig. 1:**
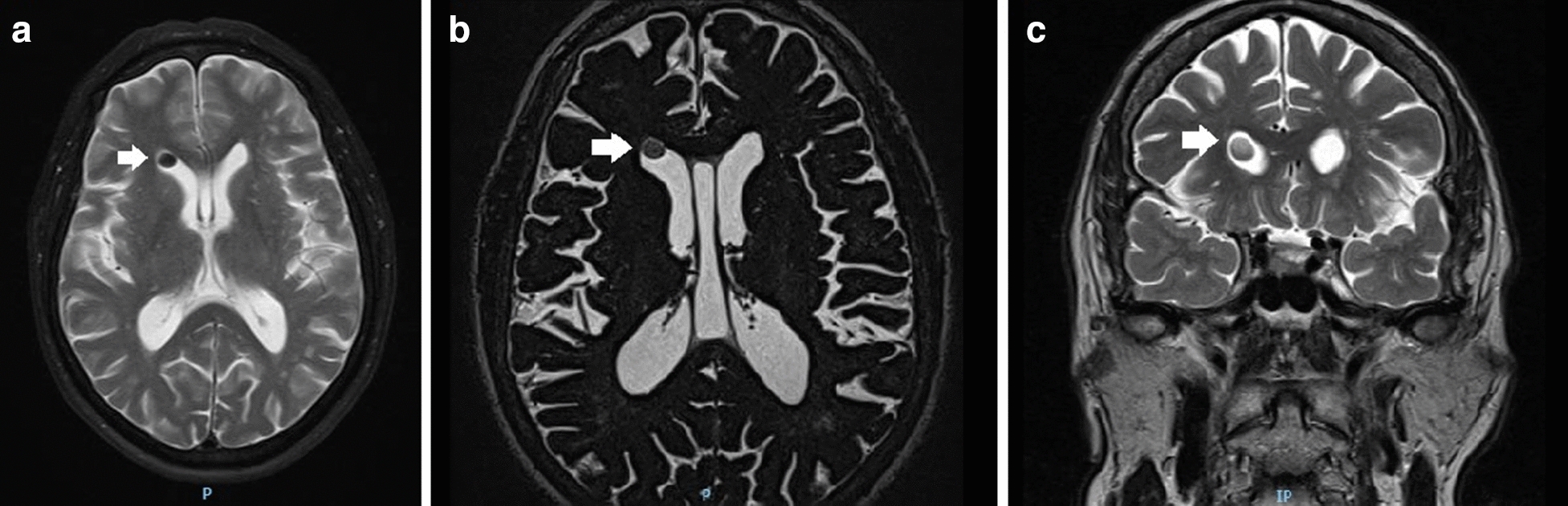
These are MRI T2 weighted images showing hypointense circular lesions within the horn of the right lateral ventricle (arrows), corresponding to migrated silicone oil droplet. **a**, **b** Showing Axial Cuts, and **c** demonstrate the same lesion in a Coronal cut

**Fig. 2:**
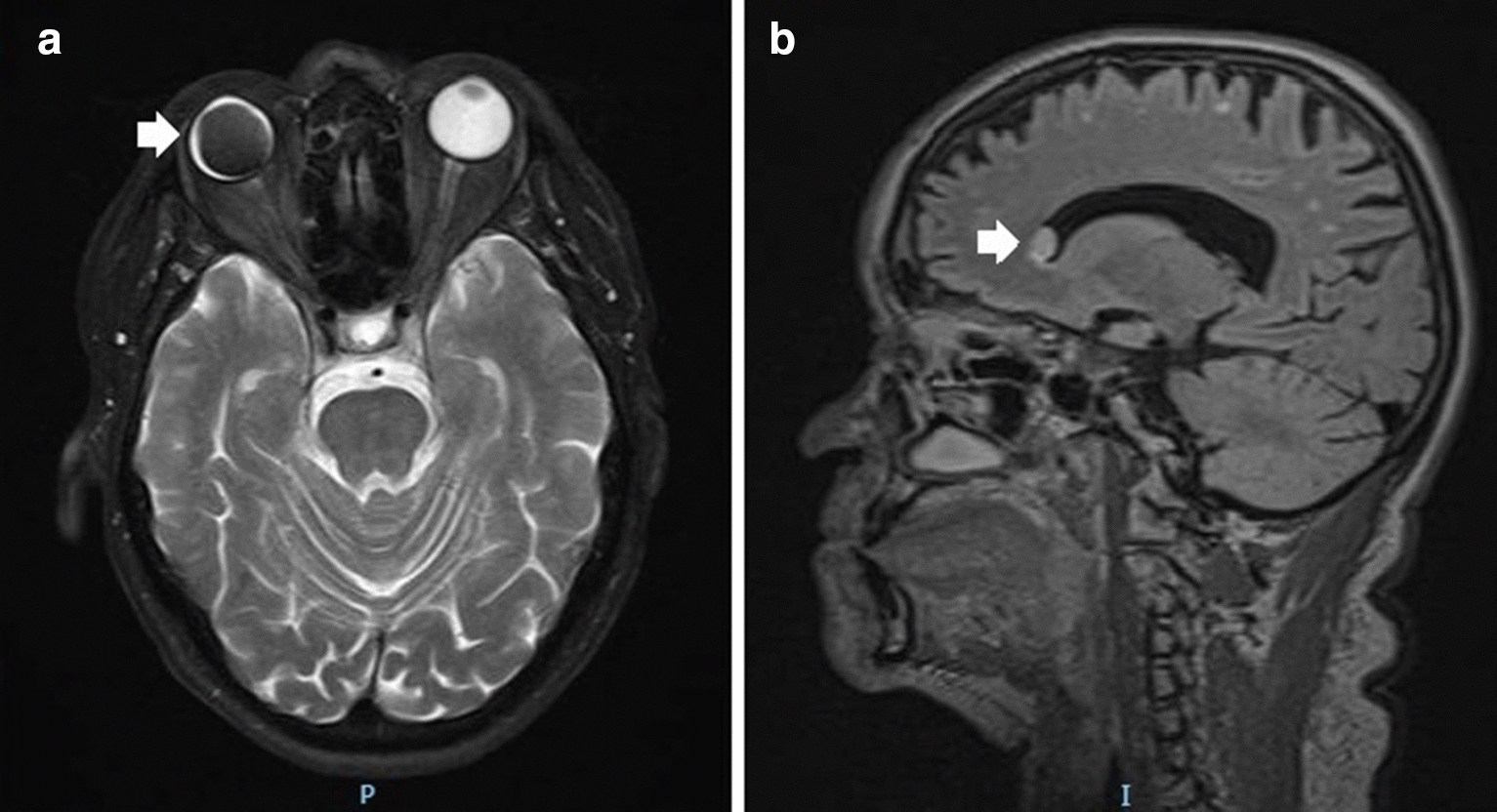
**a **The substance in the vitreous space of the right eye (SiO) showing hypointensity in a T2 weighted MRI axial cut. **b **T1 weighted MRI Sagittal cut showing hyperintense ovoid lesion on the frontal horn of the lateral ventricle (arrow), corresponding to the migrated SiO

Magnetic resonance imaging (MRI) was performed, demonstrating an ovoid lesion within the frontal horn of the right lateral ventricle. The T2 weighted images showed migrated SiO in the horn of the lateral ventricle, and it can be seen in Figs. 1 and 2a. Figure 2b shows a T1 weighted MRI Sagittal Cut image with a hyperintense ovoid lesion on the frontal horn of the lateral ventricles as well. The patient was evaluated by the neurosurgery department, in which they opted for clinical observation, oral analgesia with metamizole, and close follow-up. According to the patient, the headaches episodes diminished considerably, both in frequency and intensity. We performed panretinal photocoagulation in the left eye due to PDR, and the patient maintained a stable neuro-clinical condition until the submission of this article.

### Discussion

The migration of intravitreal silicone oil into the cerebral ventricles remains a rarely described phenomenon, and it may contribute to the misinterpretation of other brain lesions, such as ventricular hemorrhage, colloid cyst, and calcifications [9, 10]. We believe that ocular hypertension and advanced glaucoma developed after the vitreoretinal surgery in our case could have been the probable causes for the silicone oil to migrate [11].

The predominant location of migrated SiO seems to be in the lateral ventricles, which is described to be most likely asymptomatic, compared with the third or fourth ventricles. Intraventricular SiO droplets are described to potentially block the cerebrospinal fluid outflow and temporarily raise intracranial pressure. We hypothesize that this could explain the severe headaches episodes presented by our patient [12].

There are no clear indications for neurosurgical intervention to attempt intraventricular silicone removal in asymptomatic patients, even with radiographic findings, since it is described as a benign finding by several authors. Symptomatic patients are rarely described, and there is no clear consensus about the management in these cases. Close follow-up and analgesia may be reasonable in stable cases; however, there are reports of migrated SiO that caused elevated intracranial pressure, in which the headaches could only be managed with ventricularperitoneal shunt [12–14].

The radiographic appearance of intraocular SiO is distinct from other substances by MRI. In comparison to normal vitreous, SiO is hyperintense on T1-weighted images, but it has variable intensity on T2-weighted images, an effect that has been attributed to differences in oil viscosity, degree of emulsification, and MRI sequence parameters. An MRI scan in the prone position may be used to document the buoyancy of silicone due to its lower density compared to the cerebral spinal fluid, which may aid in the differential diagnosis [11].

Previous studies tried to investigate the frequency of silicone oil extraocular migration since the prevalence of this phenomenon is not well established. Grzybowski and colleagues evaluated 19 patients with MRI at a mean of 115 days after silicone oil tamponade, demonstrating no signs of extravasation [12]. However, none of the patients in the study had an underlying risk factor for oil migration, such as glaucoma, optic atrophy, or optic pit, so the subgroup with the highest risk of this complication might have been missed [5].

### Conclusions

As final considerations, the present case illustrates the importance of considering this diagnosis when encountering abnormal neuroimaging findings in a patient with a pertinent history of previous vitreoretinal surgeries. A wider knowledge of this phenomenon and intracranial silicone’s radiographic appearance may avoid unnecessary prolonged hospital stays and neurosurgical interventions.

## Data Availability

Not applicable.
